# Editorial: Molecular Mechanisms of Plant Stress Adaptation

**DOI:** 10.3390/biology15141195

**Published:** 2026-07-20

**Authors:** Wenqiang Li

**Affiliations:** College of Life Sciences, Northwest A&F University, Yangling 712100, China; wqli@nwafu.edu.cn

Global climate change has amplified both the frequency and severity of abiotic stresses including drought, salinity and extreme temperatures, while concurrently modifying pathogen population dynamics, thereby imposing unparalleled threats to agricultural crop productivity and the health of forest ecosystems. Unraveling the molecular mechanisms of plant stress adaptation is therefore not only a fundamental biological problem‌ but also a strategic priority for sustainable agriculture. This Special Issue includes ten original research papers and review articles that together show the diverse and complex ways plants sense, transmit signals, and react to environmental stresses. With a core focus on three globally critical staple food crops (rice, wheat, maize), and further covering woody perennials (oak, Phoebe) and dioecious species (mulberry), these contributions demonstrate both stress-specific pathways and shared regulatory hubs, offering a multifaceted view of plant stress adaptation.

Cold stress is a major limitation for rice production in temperate regions. In a comprehensive review, Shahzad et al. [1] summarize current knowledge on cold-responsive genes in rice (*Oryza sativa*), detailing the functional roles of CBF/DREB1 cascades, reactive oxygen species scavenging systems, and membrane lipid remodeling. Their work provides a roadmap for biotech breeding and genetic improvement of chilling tolerance. Beyond low temperature, drought and salinity remain the most widespread yield-limiting factors. Ma et al. [2] performed genome-wide identification and expression profiling of the phospholipase A (PLA) family in mulberry, revealing that specific PLA isoforms are dynamically upregulated under both drought and salt treatments, implicating lipid signaling in osmotic adjustment. In a different context, Yang et al. [3] investigated how drought severity and nitrogen addition interactively modulate seedling growth and resource-use strategies in *Quercus wutaishanica*, a keystone oak species. Their findings demonstrate that moderate nitrogen supply can alleviate drought-induced growth inhibition by enhancing water-use efficiency and photosynthetic nitrogen allocation, but excessive nitrogen intensifies stress under severe drought, highlighting the importance of balanced nutrient management in forest restoration. For salt stress, Kim et al. [4] employed integrated genomic and transcriptomic analyses in wheat roots and uncovered a two-tier adaptive strategy: a constitutive auxin biosynthetic capacity that supports basal root elongation, coupled with stress-responsive transcriptional repression that fine-tunes growth under salinity. This work effectively resolves the trade-off between maintenance of root architecture and active stress defense.

Impressively, plants commonly manage multiple overlapping stresses via central regulatory hubs. Li et al. [5] review the multifaceted roles of heat shock transcription factors (HSFs), demonstrating that these regulators extend far beyond thermotolerance to orchestrate cross-protective responses against drought, salinity, and oxidative insults. Their analysis positions HSFs as promising targets for engineering broad-spectrum stress tolerance. Complementing this, Fan et al. [6] dissect the contribution of TATA-box elements and 5′UTR sequences in rice defense signal transduction‌ upon blast fungus infection, showing that promoter architecture governs the rapid induction of pathogenesis-related genes. This regulatory layer enhances the precision of our understanding of biotic stress perception.

Achieving stress tolerance without compromising plant growth represents a core challenge in modern breeding. Several studies featured in this issue directly investigate the complex interplay between plant growth and stress resilience. Chen et al. [7] employed transcriptomics to reveal how an endophytic fungus enhances root growth and stress resistance in *Phoebe bournei*, a valuable timber tree. Fungal colonization upregulated auxin-related genes and antioxidant enzymes, simultaneously boosting biomass and tolerance to drought and low temperature. Meanwhile, the wheat root system, as shown by Kim et al. [4], already exemplifies a constitutive–inducible balance that coordinates elongation with salt defense. Interestingly, sexual dimorphism in stress responses is rarely studied, but Shi et al. [8] provide mechanistic insights into the development and maintenance of sexual dimorphism in dioecious mulberry (*Morus alba*). They identify sex-biased expression of hormone signaling and secondary metabolism genes, which may contribute to differential stress susceptibility between male and female plants, a factor with practical implications for orchard design.

Exogenous application of osmotic protectants offers a non-transgenic route to enhance stress resistance. Wang et al. [9] examined the effects of glycinebetaine on maize under drought and flooding, revealing contrasting regulatory patterns: glycinebetaine enhanced antioxidant capacity and photosynthetic efficiency under drought, whereas under flooding, it mitigated oxidative damage by modulating anaerobic respiration pathways. These results underscore the need for stress-specific application strategies. Finally, an integrative review by Aslam et al. [10] compiles rice transcriptomic and proteomic stress studies and highlights key networks and candidate genes for multi-tolerance improvement.

Overall, the works in this Special Issue apply multi-omics approaches, from genome-wide identification and transcriptomic profiling to integrated transcriptome and proteome analysis, to systematically dissect molecular mechanisms of plant stress adaptation. Spanning cis-regulatory elements, hormonal crosstalk and microbial symbiosis, these mechanistic findings not only enrich our understanding of plant stress responses, but also provide abundant molecular breeding targets and management strategies for sustainable crop and forest improvement. I sincerely thank all authors for their rigorous contributions, reviewers for their constructive comments, and the *Biology* editorial team for their reliable support. It is our expectation that these studies will push more research to turn lab discoveries into real field applications, offering simple, workable solutions for climate-adapted crop production and ecosystem care.

## Data Availability

Data available on request from the authors.
